# Catatonia Induced by First-Time Use of Synthetic Cannabinoids: A Case Report

**DOI:** 10.7759/cureus.53324

**Published:** 2024-01-31

**Authors:** Avanti Puri, Filippo Costanzo, Janny Rivera, Jean Bujdos

**Affiliations:** 1 Psychiatry, Arnot Ogden Medical Center, Elmira, USA; 2 Psychiatry, Cleveland Clinic Akron General, Akron, USA; 3 Psychiatry, St. Luke's Health Network, Lehighton, USA

**Keywords:** cannabinoids, cannabis-induced catatonia, synthetic cannabis, catatonia, case report

## Abstract

We present the case of a 32-year-old woman who developed life-threatening catatonia in the setting of synthetic cannabis use. She was treated with high doses of lorazepam (up to 26 mg) and eventually transferred to receive electroconvulsive therapy (ECT). Synthetic cannabis poses a unique risk as it is widely available, difficult to regulate, and with adverse effects that are not well understood due to the presence of ever-changing chemical compounds. In this case report, we present one of the first cases of catatonia induced by first-time synthetic cannabinoids with no previous history of cannabis use disorder.

## Introduction

Compared to cannabis, synthetic cannabis has more severe and less predictable neuropsychiatric consequences. This is thought to be in part because synthetic cannabis is a full agonist of endocannabinoid receptors CB1 and CB2, whereas tetrahydrocannabinol (THC) is only a partial agonist [1]. Synthetic cannabis is colloquially known as K2, spice, and kush and is sometimes marketed as "herbal incense" or "potpourri." Its use is increasing at an alarming rate, likely due to its wide availability, perceived legality, and difficulty to detect in urine drug screens [2-4]. In 2012, 26 types of synthetic cannabis were permanently listed as Schedule 1-controlled substances, defined as drugs with no currently accepted medical use and a high potential for abuse. Despite this and the fact that 43 states have taken action to control the use of synthetic cannabis, its regulation remains a challenge [3]. Manufacturers try to get around these laws by creating new products with different ingredients or by labeling them “not for human consumption.” The diverse array of chemical compounds found in synthetic cannabis products not only complicates regulation but also makes it difficult to predict its adverse effects [1-3]. One study found that patients who used synthetic cannabis had more severe psychotic symptoms, received higher doses of antipsychotics, and had longer inpatient hospitalizations compared to those who used cannabis [4].

The endocannabinoid system plays a widespread role in neuromodulation, from synaptic plasticity to central nervous system development, and its disruption can lead to schizophrenia [5]. Repeated use of cannabinoids can result in tolerance and produce withdrawal symptoms that may include seizures, which our patient experienced after three days of synthetic cannabis use [6]. Higher affinity at the CB1 receptor in synthetic cannabis leads to the neurotransmitter imbalance thought to be responsible for catatonia: glutamate hyperactivity and gamma-aminobutyric acid (GABA) hypoactivity [1,5,6]. Further, the location of CB1 receptors is found throughout the brain but is highly concentrated in the basal ganglia, neocortex, and hippocampus - regions that play a role in catatonia [7].

Catatonia is said to be underdiagnosed due to its wide range of presentations and diverse differential including not only psychiatric etiologies but also neurologic, autoimmune, and substance-induced causes [8]. In previous case reports of catatonia induced by synthetic cannabinoids, heavy use was reported for at least two weeks, along with periodic use of cannabis [1,9,10]. In this case report, we illustrate a case of treatment-resistant catatonia induced by first-time use of synthetic cannabis.

## Case presentation

A 32-year-old Caucasian female with a psychiatric history of post-traumatic stress disorder was brought to the emergency room by her husband for three days of minimal speech and appetite, markedly flattened affect, and occasional paranoid statements. She was not prescribed any psychiatric medications, and her husband denied regular substance use; however, he reported that she had taken three 25-mg delta-10 synthetic cannabis edibles for musculoskeletal pain before symptom onset. Medical workup in the emergency room was negative, except for urine toxicology showing THC.

She was admitted to an inpatient psychiatric unit due to these sudden behavioral changes. Three days later, she experienced a suspected seizure with severe tongue laceration, leading to transfer to a medical unit where she was started on IV levetiracetam. On day 5, she was evaluated by the psychiatry consult team. She was unresponsive to pain and voice commands, with eyes closed and constant non-purposeful motor unrest, thrashing her arms around. She was initially thought to be in a post-ictal state but had no improvement despite 48 hours of treatment with an anti-epileptic. A brain MRI was ordered, and lorazepam 2 mg IV was administered due to movements. Minutes later, she exhibited a marked response, sitting up and conversing, with an end to the constant movements. Ultimately, the MRI could not be completed, but the patient did receive an EEG that showed cerebral swelling, which was thought to be consistent with the post-ictal state.

A provisional diagnosis of catatonia was made, and the patient slowly improved with scheduled IV lorazepam. Lorazepam was gradually titrated to 26 mg daily in divided doses (8, 8, and 10 mg) as electroconvulsive therapy (ECT) was not available, and the patient was still far from her cognitive baseline and did not eat or drink. On day 12, IV nutrition (TPN) was initiated. Olanzapine 5 mg was started on day 20 for fearful, paranoid statements (i.e., waking up and being afraid of her feet). Transfer to an outside facility for ECT was considered due to partial response to lower doses of lorazepam; however, no facility was readily available, and she continued to improve on increasing doses of lorazepam. She tolerated transitioning from IV to oral lorazepam with no issue, with a 1:1 conversion. Due to incomplete response and the high dose and length of lorazepam taper required, she was transferred on day 30 to an outside facility for ECT while continuing lorazepam.

## Discussion

In this case report, we present one of the few cases of catatonia induced by synthetic cannabinoids with no previous history of cannabis use disorder [1,9,10]. This case illustrates the reality that after as little as three doses of a synthetic cannabinoid product, one may develop treatment-resistant catatonia.

In Palma-Álvarez et al.'s 2021 review of 11 case reports documenting 14 patients with cannabis-induced catatonia, 10 of 14 patients had psychotic symptoms and were started or continued on an antipsychotic [1]. This finding was helpful for diagnostic clarity in our case, and our patient’s psychosis improved as the motor symptoms of her catatonia improved. Another study of 148 catatonic patients across different diagnostic groups found that 75% experienced psychotic symptoms. They also described patients “recalling having been in a state of intense fear,” similar to our patient's description [11].

This case illustrated the potency of synthetic cannabinoids in association with treatment-resistant catatonia. Our patient did not have significant improvement in catatonia until lorazepam was titrated to 26 mg daily, and even then, she was not at her baseline. Although not unheard of, 26 mg/day is close to the upper limit of what has been described in the treatment of catatonia, with established texts citing dosing up to lorazepam 30 mg [1,4]. In the review by Palma-Álvarez et al., significantly lower doses of lorazepam were needed to achieve remission of catatonia, with 2 mg lorazepam used to treat six patients, 4 mg for four patients, 5 mg for one patient, 6 mg for one patient, and 1 mg for one patient. Two of the 14 patients in previous case reports of cannabis-induced catatonia required ECT for full remission of catatonia [1].

While our patient had recovered to near baseline at 26 mg of lorazepam, an important consideration of this case is the feasibility of outpatient management, considering a high dose of lorazepam could require a three- to six-month taper to prevent relapse [12,13]. Initiation of ECT is recommended in cases with a lack of or partial response to benzodiazepines after one week [1,4,14]. There is limited data on the rates and availability of ECT use in the United States, though anecdotally, for the most part, only facilities in major cities have access to ECT. This necessitates augmentation and continued treatment of lorazepam while a transfer for ECT is considered and facilitated. High doses of lorazepam can complicate the course of treatment as the medication requires significant reduction and tapering of the medication to not interfere with the ECT process [12,13].

## Conclusions

In conclusion, this is a case in which synthetic cannabis induced severe, treatment-resistant catatonia. While ECT remains the gold standard of treatment, the use of high-dose lorazepam is a reasonable treatment option, especially when facing difficulties in transferring to a facility for ECT. Possible difficulties include a lack of bed availability, COVID restrictions, and a family’s hesitation to transfer, among others. It is important to weigh these concerns against those of high-dose lorazepam treatment, such as the lengthy benzodiazepine taper, outpatient provider resistance in accepting patients, a pharmacist’s resistance to filling the script, and even insufficient stock of lorazepam at the pharmacy. This all needs to be taken into account when making a treatment decision in a facility without access to ECT. Finally, given the wide availability and difficulties in regulation of synthetic cannabis, it is paramount for physicians to counsel patients on its unknown adverse effects and the possibility of life-threatening consequences such as catatonia.
